# Supplementary material for: FastqPuri: high-performance preprocessing of RNA-seq data
Source: BMC Bioinformatics. 2019 May 3;20:226. doi: 10.1186/s12859-019-2799-0 (PMC6500068; doi:10.1186/s12859-019-2799-0)
Supplement: Supplementary file 2 — Archive of FastqPuri. Archive containing all files needed to install and run FastqPuri v1.0.6. Date stamp March 22, 2019. (GZ 47,819 kb) [file 12859_2019_2799_MOESM2_ESM.gz › FastqPuri-1.0.6/html/trimDS_8c.html]

FastqPuri: src/trimDS.c File Reference


FastqPuri


- src

Functions |
Variables

trimDS.c File Reference

trim adapters from double stranded data
More...

`#include <stdio.h>`  
`#include <stdlib.h>`  
`#include <string.h>`  
`#include "trimDS.h"`  
`#include "Lmer.h"`  
`#include "trim.h"`  
`#include "struct_trimFilter.h"`

Include dependency graph for trimDS.c:

|  |  |
| --- | --- |
| Functions | |
| DS\_adap | init\_DSadap (char \*ad1, char \*ad2, int L1, int L2) |
|  | initialization of a DS\_adap structure More... |
|  | |
| static void | pack\_reads (DS\_adap \*ptr\_DSad, Fq\_read \*r1, Fq\_read \*r2) |
|  | pack reads with process\_seq so that we can compute the edit distance between two subsequences. Read 1 will be packed once in the forward direction and Read 2 twice in the reverse direction with a shift of half a byte. More... |
|  | |
| static int | QtrimDS (Fq\_read \*r1, Fq\_read \*r2, int L) |
|  | trims both reads reducing them to a length L. More... |
|  | |
| static double | obtain\_scoreDS (Fq\_read \*r1, int pos1, Fq\_read \*r2, int pos2) |
|  | obtains the score when comparing two subsequences of extended r1 and extended r2, starting in pos1 and pos2 respectively. The score is computed by adding log\_10(4) when a match is observed and subtracting Q/10.0 when a mismatch is observed, with Q being the quality value. If there is a mismatch in a region where both read 1 and read 2 have qualities associated to the nucleotide under consideration, then the maximum of the quality values is subtracted. More... |
|  | |
| static int | alignDS\_uint64 (Fq\_read \*r1, Fq\_read \*r2) |
|  | try to find adapter remnants in the reads r1 and r2 by aligning their extended versions (adapter1 + r1) vs rev\_comp(adapter2 + r2). More... |
|  | |
| int | trim\_adapterDS (DS\_adap \*ptr\_DSad, Fq\_read \*r1, Fq\_read \*r2) |
|  | trim the sequences, discard them or keep them unchanged depending on them having adapters remnants. More... |
|  | |

|  |  |
| --- | --- |
| Variables | |
| uint8\_t | fw\_1B [256] |
|  | |
| uint8\_t | bw\_1B [256] |
|  | |
| Iparam\_trimFilter | par\_TF |
|  | |

## Detailed Description

trim adapters from double stranded data

Author
:   Paula Perez paula.nosp@m.pere.nosp@m.zrubi.nosp@m.o@gm.nosp@m.ail.c.nosp@m.om

Date
:   05.10.2017

## Function Documentation

## ◆ alignDS\_uint64()

|  |  |  |  |
| --- | --- | --- | --- |
| static int alignDS\_uint64 | ( | Fq\_read \* | *r1*, |
|  |  | Fq\_read \* | *r2* |
|  | ) |  |  |

static

try to find adapter remnants in the reads r1 and r2 by aligning their extended versions (adapter1 + r1) vs rev\_comp(adapter2 + r2).

Parameters
:   |  |  |
    | --- | --- |
    | r1 | pointer to Fq\_read for read 1 |
    | r2 | pointer to Fq\_read for read 2 |

Returns
:   0 if the read is to be discarded, 1 if left as is, 2 if trimmed. Reads are trimmed if 2 is returned.

## ◆ init\_DSadap()

|  |  |  |  |
| --- | --- | --- | --- |
| DS\_adap init\_DSadap | ( | char \* | *ad1*, |
|  |  | char \* | *ad2*, |
|  |  | int | *L1*, |
|  |  | int | *L2* |
|  | ) |  |  |

initialization of a DS\_adap structure

Parameters
:   |  |  |
    | --- | --- |
    | ad1 | adapter 1 sequence |
    | ad2 | adapter 2 sequence |
    | L1 | adapter 1 sequence length |
    | L2 | adapter 2 sequence length |

Returns
:   initialized DS\_adap structure

## ◆ obtain\_scoreDS()

|  |  |  |  |
| --- | --- | --- | --- |
| static double obtain\_scoreDS | ( | Fq\_read \* | *r1*, |
|  |  | int | *pos1*, |
|  |  | Fq\_read \* | *r2*, |
|  |  | int | *pos2* |
|  | ) |  |  |

static

obtains the score when comparing two subsequences of extended r1 and extended r2, starting in pos1 and pos2 respectively. The score is computed by adding log\_10(4) when a match is observed and subtracting Q/10.0 when a mismatch is observed, with Q being the quality value. If there is a mismatch in a region where both read 1 and read 2 have qualities associated to the nucleotide under consideration, then the maximum of the quality values is subtracted.

Parameters
:   |  |  |
    | --- | --- |
    | r1 | pointer to Fq\_read for read 1 |
    | pos1 | position to start comparing in read 1, starting from 5' end of the extended sequence (adapter 1 + read 1) |
    | r2 | pointer to Fq\_read for read 2 |
    | pos2 | position to start comparing in read 2, starting from 3' end of the extended sequence (adapter 2 + read 2) |

Returns
:   score associated to the comparison of the two strings

## ◆ pack\_reads()

|  |  |  |  |
| --- | --- | --- | --- |
| static void pack\_reads | ( | DS\_adap \* | *ptr\_DSad*, |
|  |  | Fq\_read \* | *r1*, |
|  |  | Fq\_read \* | *r2* |
|  | ) |  |  |

static

pack reads with process\_seq so that we can compute the edit distance between two subsequences. Read 1 will be packed once in the forward direction and Read 2 twice in the reverse direction with a shift of half a byte.

Parameters
:   |  |  |
    | --- | --- |
    | ptr\_DSad | pointer to DS\_adap structure, contains adapters sequences |
    | r1 | pointer to Fq\_read for read 1, packed sequence stored here. |
    | r2 | pointer to Fq\_read for read 2, packed sequences stored here. |

## ◆ QtrimDS()

|  |  |  |  |
| --- | --- | --- | --- |
| static int QtrimDS | ( | Fq\_read \* | *r1*, |
|  |  | Fq\_read \* | *r2*, |
|  |  | int | *L* |
|  | ) |  |  |

static

trims both reads reducing them to a length L.

Parameters
:   |  |  |
    | --- | --- |
    | r1 | pointer to Fq\_read for read 1 |
    | r2 | pointer to Fq\_read for read 2 |
    | L |  |

Returns
:   2 (we are trimming the sequences)

## ◆ trim\_adapterDS()

|  |  |  |  |
| --- | --- | --- | --- |
| int trim\_adapterDS | ( | DS\_adap \* | *ptr\_DSad*, |
|  |  | Fq\_read \* | *r1*, |
|  |  | Fq\_read \* | *r2* |
|  | ) |  |  |

trim the sequences, discard them or keep them unchanged depending on them having adapters remnants.

Returns
:   0 if the read is to be discarded, 1 if left as is, 2 if trimmed. Reads are trimmed if 2 is returned.

## Variable Documentation

## ◆ bw\_1B

uint8\_t bw\_1B[256]

global variable. Lookup table.

## ◆ fw\_1B

uint8\_t fw\_1B[256]

global variable. Lookup table.

## ◆ par\_TF

Iparam\_trimFilter par\_TF

global variable. Input parameters.

global variable: Input parameters trimFilter.

global variable: Input parameters of makeTree.


---

Generated on Mon Mar 19 2018 23:42:01 for FastqPuri by  

 1.8.14
